# Editorial: Economic and financial issues in the post-COVID-19 world: Implications and role of public health

**DOI:** 10.3389/fpubh.2022.1126501

**Published:** 2023-01-11

**Authors:** Giray Gozgor, Chi Keung Marco Lau

**Affiliations:** ^1^Faculty of Political Sciences, Istanbul Medeniyet University, Istanbul, Turkey; ^2^School of Business, Teesside University, Middlesbrough, United Kingdom

**Keywords:** the COVID-19 pandemic, the post-COVID-19 era, public health, economic issues, financial issues

The COVID-19 crisis requires an integrated effort from governments, institutions, organizations, and communities to attack this crisis. The COVID-19 pandemic has imparted several economic and financial shocks to society. We believe that the nature of work everywhere will be transformed in the aftermath of this global pandemic; for example, online working from home is increasingly adopted by companies wishing to reduce fixed costs and minimize organizational risk. Also, there should be significant changes in lifestyle and access to basic infrastructures, such as energy and transportation systems. Therefore, governments should promote green energy and transportation in the post-COVID-19 world. This issue can also change the pollution levels in the 2020s. Finally, the COVID-19 pandemic provided a significant inequality between developing and advanced economies. Decreasing inequality (including health-access inequality) should also be essential, especially in the United States and the United Kingdom's free-market economies.

Given this background, this Research Topic aims to investigate the effects of the COVID-19 pandemic on leading economic and financial indicators in developing and developed economies. Each paper also aimed to discuss the potential implications for the post-COVID-19 era.

This Research Topic covers 46 papers on COVID-19 and the post-COVID-19 era.

The first paper finds that pandemic uncertainty decreases human capital in the panel dataset of 122 countries from 1996 to 2019 (Sun, Cai et al.). It is also shown that the COVID-19 pandemic has had a critical incentive effect on knowledge collaboration (Zhou, Chen et al.). It is indicated that Artificial intelligence technology can alleviate the plights of firms in the post-pandemic era (Lu, Wijayarantna et al.). Investors' uncertainty sentiment is negatively related to the Chinese stock return from 2 March 2020 to 2 March 2021 (Xu, Zhang et al.). Another paper discusses how governments should evaluate their pandemic policies to determine their effectiveness (Li, Fang et al.). The paper also measured China's market power in medical product exports, including the COVID-19 era, from 1992 to 2020 (Wu et al.). It is observed that the negative impact of the COVID-19 pandemic on Corporate Social Responsibility efficiency from 2012 to 2020 in China (Chen et al.). A significant convergence of the vaccination rates in seven clubs across 30 Organization for Economic Co-operation and Development countries (Xu, Lau et al.). It is shown the significant role of China's monetary policy implementation on the industrial structure during the COVID-19 pandemic (Zhou, Wang et al.). It is stated that the significant impact of the COVID-19 pandemic on consumer behavior in China's stock market (Lu, Zhu et al.). Another paper discussed the role of lockdowns on economic losses during the COVID-19 pandemic (Zhang, You et al.). It is shown that the digital economy significantly positively affects economic growth in the Chinese regions, and this may be a valuable finding for digital transformation during the COVID-19 pandemic (Zhang, Zhao et al.). It is observed that pandemics-related uncertainty decreased fertility rates in the panel dataset of 126 countries from 1996 to 2019 (Wang, Gozgor et al.). It is found that the COVID-19 pandemic enhanced the congestion effect of tourism industry agglomeration on total factor productivity in China (Wang and Ma). It is stated that the COVID-19 pandemic has promoted job opportunities in the healthcare sector (Bachmann et al.). It is discussed that China's carbon Emissions Trading System would be necessary for carbon reductions after the post-pandemic era (Luo et al.).

Furthermore, it is found that bilateral investment agreements improved the efficiency of the contract execution panel data of 73 developing countries from 2005 to 2019 (Yu et al.). It is shown that digital finance promoted economic development in the Chinese regions from 2013 to 2020 (Ye et al.). It is observed that the market mechanism motivates enterprises' green technology innovation during pandemics (Gong and Dai). It is found that the COVID-19-related uncertainty increased the corporate tax rates in the panel dataset of 113 countries from 2020 to 2021 (Li, Li et al.). It is indicated that listed private firms in China became more valuable with the “black swan” events, such as the COVID-19 pandemic (Li, Ma et al.). It is observed that public health emergencies hurt insurance companies' stock returns (Shang et al.).

On the other hand, the escalating impact of the COVID-19 pandemic on China's stock price volatility was found in the daily data from July 2020 to October 2021 (Jin). It demonstrated the negative impact of the COVID-19 pandemic on Chinese cross-border retail enterprises. It is found that the COVID-19 pandemic significantly negatively affects the stock market returns of cultural industries (Zhang, Ji et al.). It is indicated that the COVID-19 pandemic has negatively affected economic performance in panel data of 14 prefecture-level cities in Hunan from 2015 to 2020 (Pan et al.). It is also discussed the policies related to the COVID-19 pandemic crisis management of the Spanish healthcare sector (Sánchez-Bayón et al.). It is found that global pandemics sentiment positively affects the returns of the global art market (Wang). It is stated that green finance is the key to the Chinese economy from the recovery from the COVID-19 pandemic (Hu et al.). It is indicated that the industrial sector would be more critical during the post-COVID-19 pandemic era to enhance global economic activity (Ma, Shum et al.). It is observed that the COVID-19 pandemic decreased financial flexibility and firm performance in the listed hotel firms on Taiwan Stock Exchange (Zhang, Chang et al.).

Meanwhile, It was found that foreign direct investments and international trade increased income inequality in China, and the data included the COVID-19 pandemic era (Cheong et al.). It is shown that policy uncertainty in China increases the fluctuation of exports in the country from January 2002 to April 2021 (Hu and Liu). Another paper discussed the role of supply chain distortions in the COVID-19 pandemic by focusing on the case of China (Ma, Wang et al.). It is found that the digital economy increased China's cultural tourism integration during the COVID-19 pandemic (Li, Liang et al.). It also stated the negative impact of pandemics on Chinese firms' corporate innovation behavior (Zhang, Hu et al.). It is indicated that stringency measures are negatively related to the COVID-19 pandemic outcomes in the United States from 11 March 2020 to 13 August 2021 (Sun, Kwek et al.). It is also observed that the digital economy increased economic development in the regions of China, and the data included the COVID-19 pandemic era (Xu, Qian et al.). Another paper used the data in China from January 2015 to April 2021 and found the significant role of the COVID-19 pandemic on the effectiveness of the monetary policy (Song et al.). Another paper discussed the impact of the COVID-19 pandemic on working conditions using the case of survey data for dentists (Chasib et al.). It provided the negative impact of the COVID-19 pandemic on life insurers in the United States (Zhang, Liao et al.). It is found that the effects of the COVID-19 pandemic are permanent in Indonesia and the Philippines but temporary in Malaysia (Shi, Zhang et al.). It is observed that the COVID-19 pandemic significantly affected revenues and openings of small leisure and hospitality firms in the United States (Lu, Shang et al.). It is also stated that the COVID-19 pandemic harmed the international trade volume between Australia and China (Shi, Cheong et al.). It is suggested that isolation and quarantine policies as the most cost-effective intervention during the COVID-19 pandemic (Wang, Shi et al.). It is also shown that the COVID-19 pandemic negatively affected Malaysia's economic indicators (Sun, Li et al.).

In conclusion, this Research Topic shows the significant effects of the COVID-19 pandemic on various economic and financial indicators in different countries, regions and markets. It also discusses the potential implications for the post-COVID-19 era.

## Author contributions

GG and CL: writing the editorial. All authors contributed to the article and approved the submitted version.

